# Teratoid Wilms Tumor and Classical Wilms Tumor: A Retrospective 10-Year Single-Center Study and Literature Review

**DOI:** 10.3389/fsurg.2021.781060

**Published:** 2022-02-02

**Authors:** Wei Wu, Yibo Wu, Weijue Xu, Jiangbin Liu, Zhibao Lv

**Affiliations:** Department of General Surgery, Shanghai Children's Hospital, Shanghai Jiao Tong University, Shanghai, China

**Keywords:** Wilms tumor, teratoid, nephroblastoma, prognosis, histopathology

## Abstract

**Background:**

One of the most prevalent forms of renal tumors detected among pediatric patients is the Wilms tumor (WT). Teratoid WT is a rare WT subclassification that is characterized by teratoma-like characteristics that include the features of many diverse tissue categories. Less than 70 teratoid Wilms tumor (TWT) cases have been explained up to now.

**Methods:**

Between 2010 and 2020, patients with classical WT and TWT admitted to our hospital were included in this study. Clinicopathological characteristics, intraoperative findings, histopathological parameters, and prognostic outcomes were then compared between classical WT and TWT. To compare these variables, TWT and WT cases were matched at a 1:3 ratio.

**Results:**

A total of 67 total WT cases, i.e., five diagnosed with TWT, were enrolled. While no significant differences in analyzed variables were detected between these groups, tumor volumes were notably larger in the TWT group relative to the classical WT group (203.30 ± 109.89 vs. 104.30 ± 66.97 cm^3^) despite similar tumor weight values in both groups (471.00 ± 80.65 vs. 432.67 ± 109.25 g). As for five patients diagnosed with TWT, all were alive during the follow-up, while one of them was diagnosed with pelvic metastasis.

**Conclusions:**

This study is the first to our knowledge to have reported on the incidence of TWT among Chinese children, and our results preliminarily suggest that a combination of surgery and chemotherapy may be appropriate for the treatment of patients with WT, although prognostic outcomes varied substantially among patients with different stages of the disease. TWT tumor density may be lower than classical WT tumor density. Further research regarding the basic biological characteristics of TWT and relevant theranostic markers associated with this tumor type is warranted to better guide the development of individualized treatments for this rare cancer type.

## Introduction

One of the most prevalent forms of renal tumors detected among pediatric patients is Wilms tumor (WT) (1), accounting for six and 95% of total pediatric cancers and pediatric kidney tumors, respectively (2). Both tumor stage and histologic subtype are key prognostic factors in patients with WT (3). However, WT clinical characteristics are often similar to those of other rare renal tumor subtypes, necessitating careful differentiation to guide appropriate patient diagnosis and treatment (4, 5).

One of the rare forms of heterologous nephroblastoma is teratoid WT (TWT), which was first explained in 1984 by Variend et al. (6). TWT tumors are generally triphasic, exhibiting blastemal, stromal, and epithelial cell types, with other cell and tissue types, such as mucinous epithelial tissue, neurogenic tissue, osteoid tissue, adipose tissue, and cartilage, also being present in some cases. In 1988, Fernandes et al. (7) suggested that TWT diagnosis should be dependent on a <50% heterogeneous component, while the Beckwith criteria state that renal teratomas must be fully contained within the renal capsule with evidence of both a renal component and other tissue types. Given the rarity of TWT, its pathogenesis remains the subject of debate, although these heterologous tissues most likely originate from a primitive metanephric blastema (8).

Up to now, just 62 TWT cases have been explained in the English language articles, with none having been reported in China. Herein, 67 cases with WT treated at our hospital from 2010 to 2020, i.e., five cases with TWT, were retrospectively evaluated, with a particular focus on individual TWT cases with respect to their clinical presentation, preoperative findings, chemotherapeutic treatment, histopathological characteristics, and postoperative treatment and associated outcomes, which may deepen our understanding of this rare condition.

## Materials and Methods

### Patient Enrollment

From December 2010 to December 2020, 67 total cases with WT were evaluated in the hospital and were incorporated in the present survey. The treatment method is mainly based on the Children's Oncology Group (COG) and the International Society of Pediatric Oncology (SIOP). A retrospective analysis of patient clinicopathological findings, pre- and postoperative chemotherapeutic treatment strategies, intraoperative achievements histopathological findings, and prognostic outcomes was conducted. Patient follow-up was conducted every 3 months, with abdominal ultrasound scans being performed every 6 months for the first 2 years.

### WT and TWT Diagnosis and Assessment

Cases with TWT were diagnosed based upon histopathologic examination results demonstrating a >50% heterogeneous component. These tumors typically exhibit a prominent mature adipose tissue component composed of blastemal, epithelial, and stromal cell categories and may also exhibit other characteristics of other tissues, such as neurogenic tissue, cartilage, smooth muscle, osteoid tissue, squamous tissue, and mucinous epithelial tissue. Final diagnoses were established after a multi-disciplinary treatment discussion (Table 1).

**Table 1:** Teratoid Wilms tumor patient information in our hospital.

**Age at** **diagnosis**	**Sex**	**Side**	**Stage**	**Surgery**	**Size (cm)**	**LOH**	**Weight (g)**	**Histology**	**WT1**	**Chemo**	**Follow-up**
4 months	M	right	I	Right radical nephrectomy	5X3X3	no	350	FH	N	No	A and W after 3 years
3 years	F	left	II	Left radical nephrectomy	8X7X7	no	540	FH	N	Yes	A and W after 9 years
2 years	F	right	IV (pulmonary, bone marrow)	Right radical nephrectomy	12X10X6	no	460	FH	P	Yes	Pelvic relapse at 2 years
3 years	F	right	IV (lung)	Right radical nephrectomy	12X7X8	no	550	FH	P	No	A and W after 3 years
3 years	F	left	II	Left radical nephrectomy	8X9 X9	no	455	FH	P	Yes	A and W after 6 months

*A and W, Alive and well; N, Negative; P, Positive. FH, Favorable Histology*.

Non-TWT cases in this study were considered to represent classical WT cases, and a definitive classical WT diagnosis was made by experienced pathologists. A 1:3 matching ratio was used when comparing data between TWT and classical WT cases to reduce the risk of bias (Tables 2, 3).

**Table 2:** Patient demographics, clinical characteristics, and prognostic outcomes in individual groups.

		**TWT**	**Classical WT**	*P-*value^*^
Number of patients		5	15	-
Age		27.20 ± 13.97140	27.87 ± 16.91	0.93
Sex	Male	1	4	0.999
	Female	4	11	
Side	Left	2	6	0.999
	Right	3	9	
Stage	I	1	0	0.106
	II	2	7	
	III	0	6	
	IV	2	2	
Volume (cm^3^)		203.30 ± 109.89	104.30 ± 66.97	0.11
LOH	Yes	0	4	0.53
	No	5	11	
Weight (g)		471.00 ± 80.65	432.67 ± 109.25	0.42
WT1	positive	3	6	0.617
	negative)	2	9	
Chemotherapy	Yes	3	14	0.140
	No	2	1	
Survival	Yes	5	15	0.999
	No	0	0	
Relapse	Yes	1	0	0.250
	No	4	15	

* *p for continuous and categorical variables were respectively computed via Student's t-tests and Fisher's exact assessment*.

*TWT, teratoid Wilms tumor; WT, Wilms tumor*.

**Table 3:** Normality tests for quantitative data by Kolmogorov-Smirnov analysis.

	**KS test parameters**	**TWT**	**Classical WT**
Number of patients		5	15
Age	KS distance	0.3356	0.1904
	*P*-value	0.068	>0.1
	Pass normality test?	Yes	Yes
Volume (cm^3^)	KS distance	0.1963	0.1012
	*P*-value	>0.1	>0.1
	Pass normality test?	Yes	Yes
Weight (g)	KS distance	0.2214	0.1343
	*P*-value	>0.1	>0.1
	Pass normality test?	Yes	Yes

*TWT, teratoid Wilms tumor; WT, Wilms tumor*.

### Statistical Assessment

To analyze all outcomes in the present exploration, R v 3.6.3 was employed. Data are given as means with SD for continuous variables, while categorical numbers are given as numbers and percentages. Normality was assessed *via* the Kolmogorov-Smirnov assessment, with data being scrutinized *via* Fisher's exact assessment and Student's *t*-tests as appropriate. A two-sided *p* < 0.05 was the threshold of significance. Event-free survival (EFS) and overall survival (OS) curves were generated using the Kaplan-Meier method and compared using the log-rank test, using the R system for Windows (version 3.5.4). We selected all of the quantitative data from the manuscript, such as age, tumor volume, and weight, and finally found that these data pass the normality test.

### Literature Review

The PubMed/NCBI database was employed to execute a literature review using the search terms “teratoid nephroblastoma” and “TWT”, leading to the identification of 62 cases reported previously (6–41) in addition to the five cases discussed in this article (Table 4). Overall, the ages and clinical findings reported for patients with TWT are similar to those for classical patients with WT. Both girls and boys exhibited an average age of 3.1 years (range: 3 months−11 years) at diagnosis, with initial presenting symptoms, i.e., abdominal pain and an abdominal mass. At the time of presentation, 30 cases demonstrated stage I/II disease, 10 cases demonstrated stage III disease with involvement of the local lymph node, seven exhibited stage IV disease, and 15 exhibited stage V bilateral disease. A total of five patients exhibited hypertension at the time of presentation, while eight exhibited congenital abnormalities, such as horseshoe kidneys, inguinal hernias, clubbed feet, Beckwith-Wiedemann syndrome, bilateral cryptorchidism, and an ectopic ureteropelvic system. In nearly whole cases, these TWT masses exhibited favorable histological findings (Table 5).

**Table 4:** Published teratoid Wilms tumor cases.

**SN**.	**Author/Year**	**Gender**	**Sex**	**Histology**	**Follow-up**
1	Variend et al. (6)	3	F	Various epithelial and mesenchymal elements.	Unknown
2	Fernandes et al. (7)	2	M	Not reported.	Died, sepsis and renal failure
3	Fernandes et al. (7)	2	M	Not reported	NED after 7 years
4	Fernandes et al. (7)	2	M	Not reported	chronic renal failure
5	Vujanic (11)	1.1	F	Fibro adipose tissue, rhabdomyoblasts, smooth muscle, cartilage, neuroepithelium, squamous, columnar and mucinous epithelium.	NED after 2 years
6	Magee et al. (12)	2.5	M	Epithelial cells, spindle cells, mature adipose tissue.	NED after 4 years
7	Magee et al. (12)	0.9	M	Squamous, mucinous columnar epithelium, mature muscle and adipose tissue.	NED after 1 year
8	Kotiloglu et al. (13)	3	F	/Mature adipose tissue, glandular and mucinous epithelium	NED after 23 months
9	Williams et al. (14)	3	F	Skeletal muscle, adipose tissue, mucus glands.	Died from extensive pulmonary metastasis
10	Ashworth et al. (15)	3	F	Mucin secreting epithelium, fibromyxoid stroma, skeletal muscle, cartilage and adipose tissue.	Relapsed at 2 months; unknown outcome
11	Paterson et al. (16)	2	M	Mature adipose tissue, skeletal muscle, connective tissue.	Unknown
12	Karaca et al. (17)	2.5	M	Squamous epithelial component (70% tumor)	Died; pulmonary relapse at 6 months
13	Bakshi et al. (18)	1.5	M	Predominantly heterologous tissues (adipose, glial, muscle, cartilage, or bone	NED after 3 years
14	Cecchetto et al. (20)	4	F	Cylindrical ciliated, cystic squamous epithelium with hair follicles, adipose tissue muscle fibers, rhabdomyoblasts.	NED after 32 months
15	Inoue (19)	0.4	M	Stratified squamous, columnar epithelium, pigmented, mature adipose, and cartilage and bone tissue.	NED after 3 years
16	Myers (21)	4.5	F	Keratinized squamous and nodules resembling epidermoid cysts (> 50% of tumor volume)	NED after 4 years
17	Koksal (22)	2.5	M	Mature adipose tissue, skeletal muscle, bone, cartilage, and neurons.	NED 16 months
18	Parikh (27)	1	M	Heterologous/ blastemal elements.	Not reported
19	Seo et al. (25)	50	M	Heterologous elements: skeletal muscle, cartilage, adipose tissue, neural tissue; squamous epithelium.	Not reported
20	Kajbafzadeh (26)	4	M	Stromal elements, cartilage, calcification, smooth muscle fibers. Few squamoid areas.	NED 9.5 years
21	Gupta (23)	4	M	Cystic wall with colon type muscular wall	NED 5 months
22	Sultan (28)	2	M	Skeletal muscles and mature fat (85% of the tumor)	NED 20 months
23	Sultan (28)	5	F	Rhabdomyoblastic, mature adipose tissue, mucin-producing columnar epithelium	Relapse followed by remission; no evidence of disease
24	Sultan (28)	1.1	F	Skeletal muscles, mature adipose tissue, and osteoid. Glandular, squamous epithelial with focal pilosebaceous	NED 9 months
25	Mukhopadhyay (29)	4	F	Mature mucous epithelium and rhabdomyoblasts.	Unknown
26	Treetipsatit (30)	0.9	M	Skeletal muscle, mature adipose tissue, bone, small islands of odontogenic epithelium	Unknown
27	Yadav (32)	2	M	Squamous with keratin pearls (~75%); adipose and glial tissue	Unknown
28	Bardesi (31)	4	M	Cysts lined by flattened, stratified squamous epithelium, keratin flakes. Focal spindle cells /smooth muscle differentiation	NED 21 months
29	Sinha (41)	2	M	Squamous epithelium; abundant keratin pearls (~75%)	NED 21 months
30	Ramani (33)	0.3	M	Skeletal muscle; stratified squamous epithelium with keratinization	Unknown
31	Ghamdi et al. (36)	2	M	multiple foci of squamous epithelium and mature adipose tissue	NED
32	Ghamdi et al. (36)	1.8	M	foci with a triphasic pattern consisting of blastemal, epithelial and mesenchymal components	NED
33	Ghamdi et al. (36)	11	F	smooth muscle elements and extensive mature epithelial glandular elements with squamous and goblet cell differentiation	NED
34	Garje et al. (38)	4	F	classic triphasic combination of blastemal, stromal, and epithelial cell types	NED 1 year
28	Ellen (37)	21	M:F12: 16		26 Alive, NED 1 Suffered relapse, alive 1 Suffered relapse, died

*NED, No evidence of disease*.

**Table 5:** Summary of teratoid Wilms tumor cases in the published literature.

**Total no**.		**62**
Age		3.1 years (3 m−11 y)
Gender	Male	28 (45.0%)
	Female	34 (55.0%)
Stage	I and/or II	30 (48.4%)
	III	10 (16.1%)
	IV	7 (11.3%)
	V	15 (24.2%)
Histology	Favorable	59 (95.2%)
	Unfavorable	3 (4.8%)
Survival	alive	58 (93.5%)
	dead	4 (6.5%)^*^
Congenital abnormalities		Hypertension; bilateral cryptorchidism; clubfeet; Beckwith-Wiedemann syndrome; horseshoe kidney; inguinal hernia; ectopic ureteropelvic system

* *Three died of renal failure and sepsis, and/or extensive pulmonary metastasis*.

## Results

### Patient Demographics, Presentation, and Prognosis

Of the 67 patients with WT in our hospital, five patients (7.64%) were diagnosed with TWT. A 1:3 matching ratio was used when comparing data between TWT and classical WT cases to reduce the risk of bias. There were no significant differences in clinicopathological variables when comparing patients with TWT and WT, although there was a clear trend toward an increase in tumor size for patients with TWT as compared to patients with WT (203.30 ± 109.89 vs. 104.30 ± 66.97 cm^3^), despite similar tumor weight values in both groups (471.00 ± 80.65 vs. 432.67 ± 109.25). Further details regarding patient demographics, clinical characteristics, and prognostic outcomes are given in Table 2.

**Figure 1:**
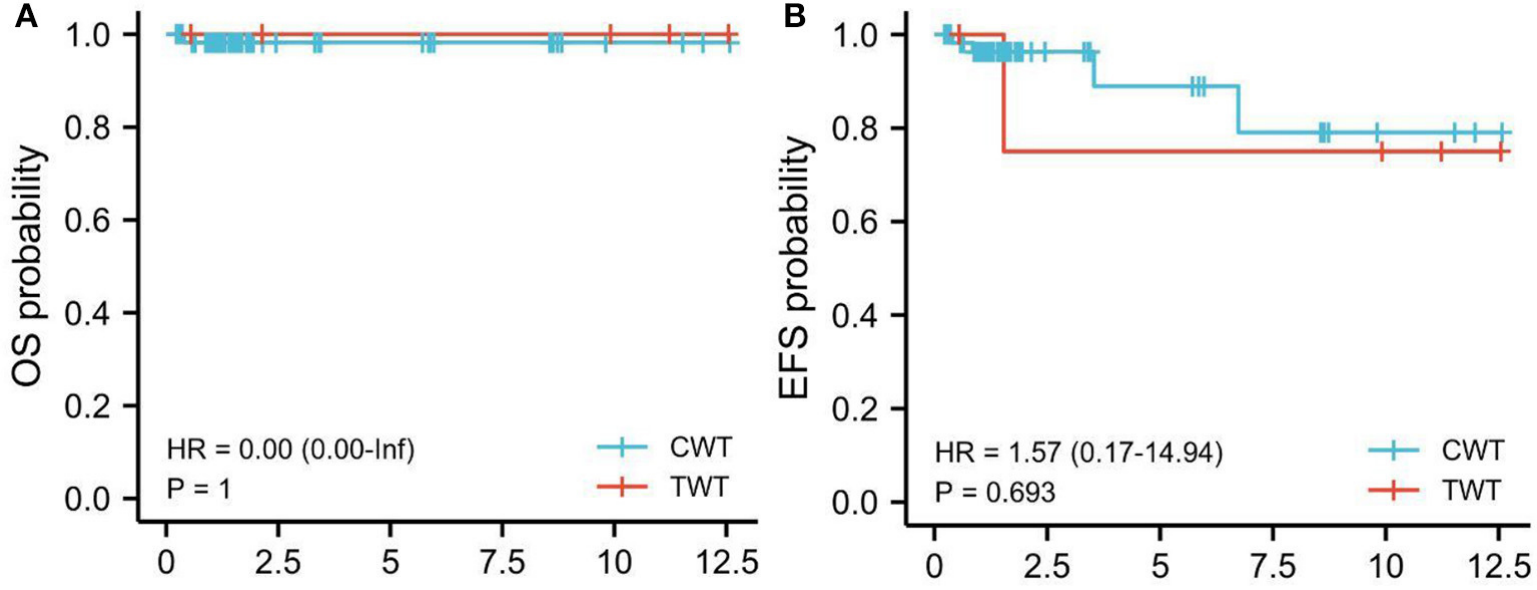
Kaplan-Meier survival analysis. EFS, TWT vs. non-TWT (CWT). Kaplan-Meier survival analysis. **(A)** OS, TWT vs. non-TWT (CWT). **(B)** EFS, TWT vs. non-TWT. Red line: TWT; Blue line: non-TWT. TWT, teratoid Wilms tumor; WT, Wilms tumor.

As to EFS and OS (62 cases with non-TWT and five cases with TWT), EFS for TWT was 80% (4/5 patients; follow-up: 6–128 months, median: 97.5 months) vs. 95% (59/62 patients; follow-up: 5–161 months, median: 76.5 months) for non-TWT. The OS for TWT was 100% (5/5 patients; follow-up: 6–128 months, median: 97.5 months) vs. 98.4% (61/62, follow-up: 5–161 months, median: 76.5.5 months) for non-TWT (Figure 1).

### Patient With TWT Clinical Details

#### Case 1

The first case was a 4-month-old boy with a right-sided abdominal mass that had been evident for 1 month. Routine preoperative analyses of serum creatinine, urea, blood urea nitrogen, and alpha-fetoprotein levels were within normal ranges. He underwent nephrectomy, yielding a sample weighing <350 g (mass weight ≈100 g) that was histologically favorable and diagnosed as a stage I tumor. Immunohistochemical staining revealed the cells of the tumor to be negative for the WT1 tumor suppressor gene. The patient was discharged without undergoing preoperative or postoperative chemotherapeutic treatment, and 9 years after initial presentation remains alive and had no recurrent disease.

#### Case 2

A 26-month-old male with a 2-week history of a distended abdomen was associated with a large abdominal mass presented. Upon physical assessment, a hard mass was palpable in the right flank without any associated tenderness. Chest and cerebral CT and bone marrow biopsy did not reveal any conclusive findings. Intraoperatively, the right kidney was found to be almost fully replaced by a tumor enveloped by a smooth capsule. No pieces of evidence of vascular invasion or inferior vena cava involvement were detected. The size of the collected nephrectomy specimen was 9 cm × 7 cm × 5 cm and its weight was 540 g. Histological examination revealed this tumor to consist of many mature epithelial glandular elements and smooth muscle elements exhibiting squamous and goblet cell-like differentiation. In addition, admixed regions of classical embryonal epithelial, blastemal, and stromal elements were evident. These cells of the tumor exhibited WT1 positivity. The patient underwent four cycles of postoperative chemotherapeutic treatment with Ifosfamide, Etoposide, and Vincristine and remained alive and disease-free as of 9 years after the surgical treating.

#### Case 3

**Figure 2:**
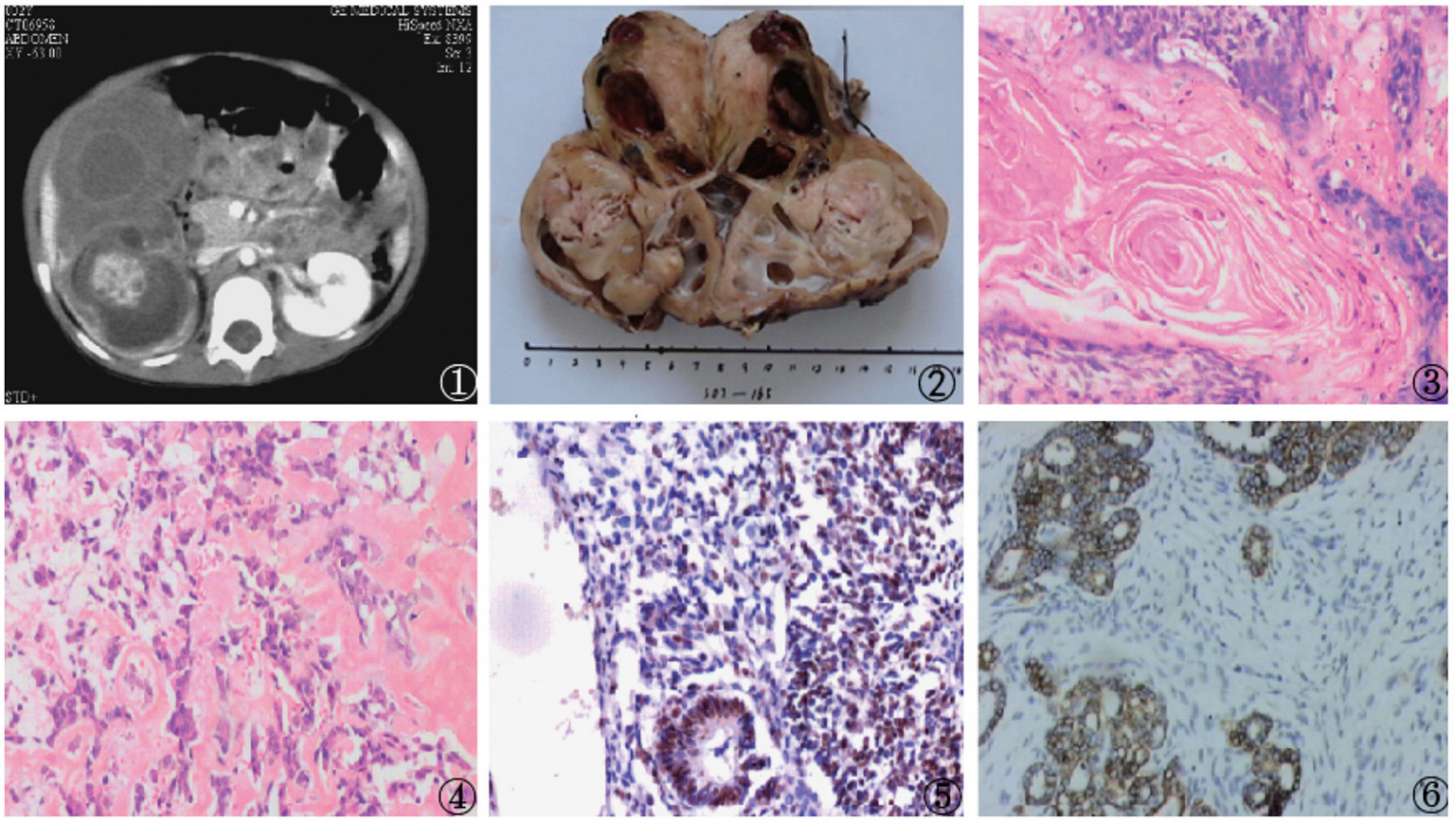
Findings from a 20-month-old girl with a right renal tumor presenting to our hospital with a background of anemia. (1) Abdominal CT scan exhibiting a large mass that had almost fully replaced the right kidney; Nephrectomy samples demonstrating the large mass that had replaced the kidney; (3–5) Histologic examination of the mass revealed a heterologous epithelium with squamous epithelial, adipose, and calcified bead characteristics (H and E, original magnification × 40); (6) Positive WT-1 immunostaining in intratumoral tubules.

A 20-month-old female with anemia (hemoglobin: 95 gm/dl) and a right-sided renal tumor was admitted to the hospital following diagnosis at a separate hospital *via* needle biopsy and exhibited metastatic stage IV disease at the time of diagnosis with bone marrow and pulmonary metastases. The patient had undergone four cycles of preoperative chemotherapeutic treatment with Ifosfamide, Etoposide, and Vincristine and exhibited a 50% reduction in renal tumor size after 8 weeks as measured *via* abdominal CT. Four weeks later, nephrectomy was performed, and neither tumors were observed within intrarenal vessels nor were lymph node metastases detected. An ovoid cystic-solid mass (9 cm × 10 cm × 9 cm) with adherent renal tissue on either side was observed, with this tumor being primarily composed of heterologous tissues (cartilage, lack of keratin, muscle, adipose, or lace-like osteoid) together with regions that were histologically consistent with classic WT (blastemal, epithelial, and stromal). Immunohistochemical analyses revealed these tumor cells to be positive for WT1. After surgery, the patient underwent four cycles of treatment with Ifosfamide, Etoposide, and Vincristine. However, pelvic implantation metastasis was observed *via* abdominal CT at the 6-month follow-up time point. The patient remains alive 2 years after the surgical treating (Figure 2).

#### Case 4

**Figure 3:**
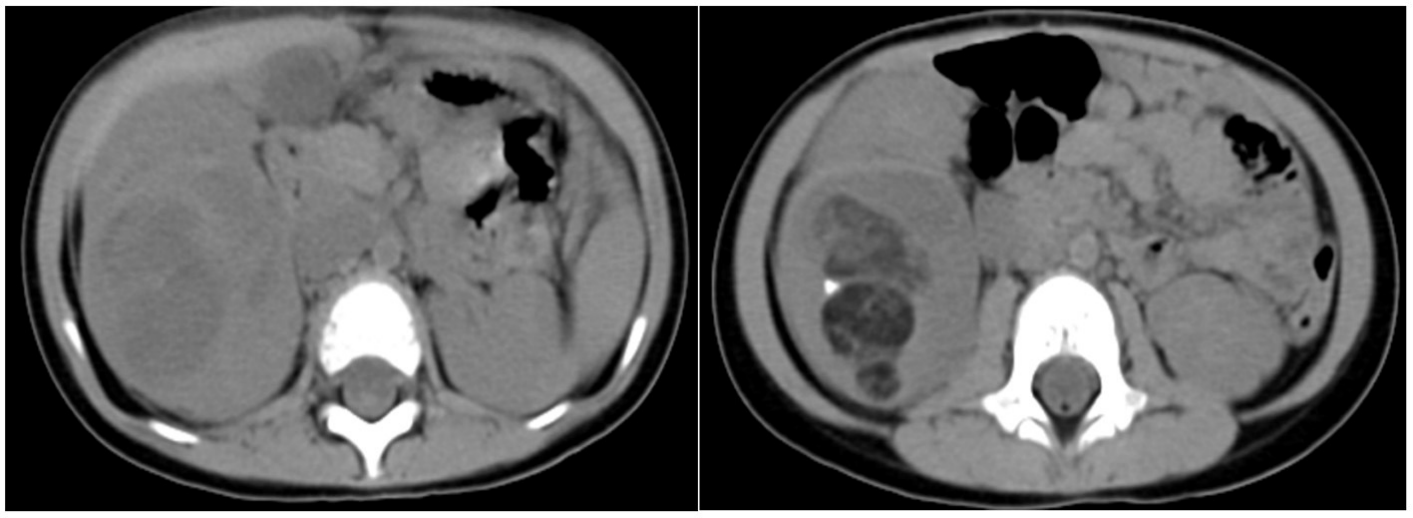
Findings in a 38-month-old girl presenting with abdominal pain for 1 week. Contrast-enhanced abdominal CT images revealed a heterogeneous mass in the middle and lower portions of the right kidney (8 cm × 8 cm × 9 cm) with imaging findings consistent with fat or hair.

**Figure 4:**
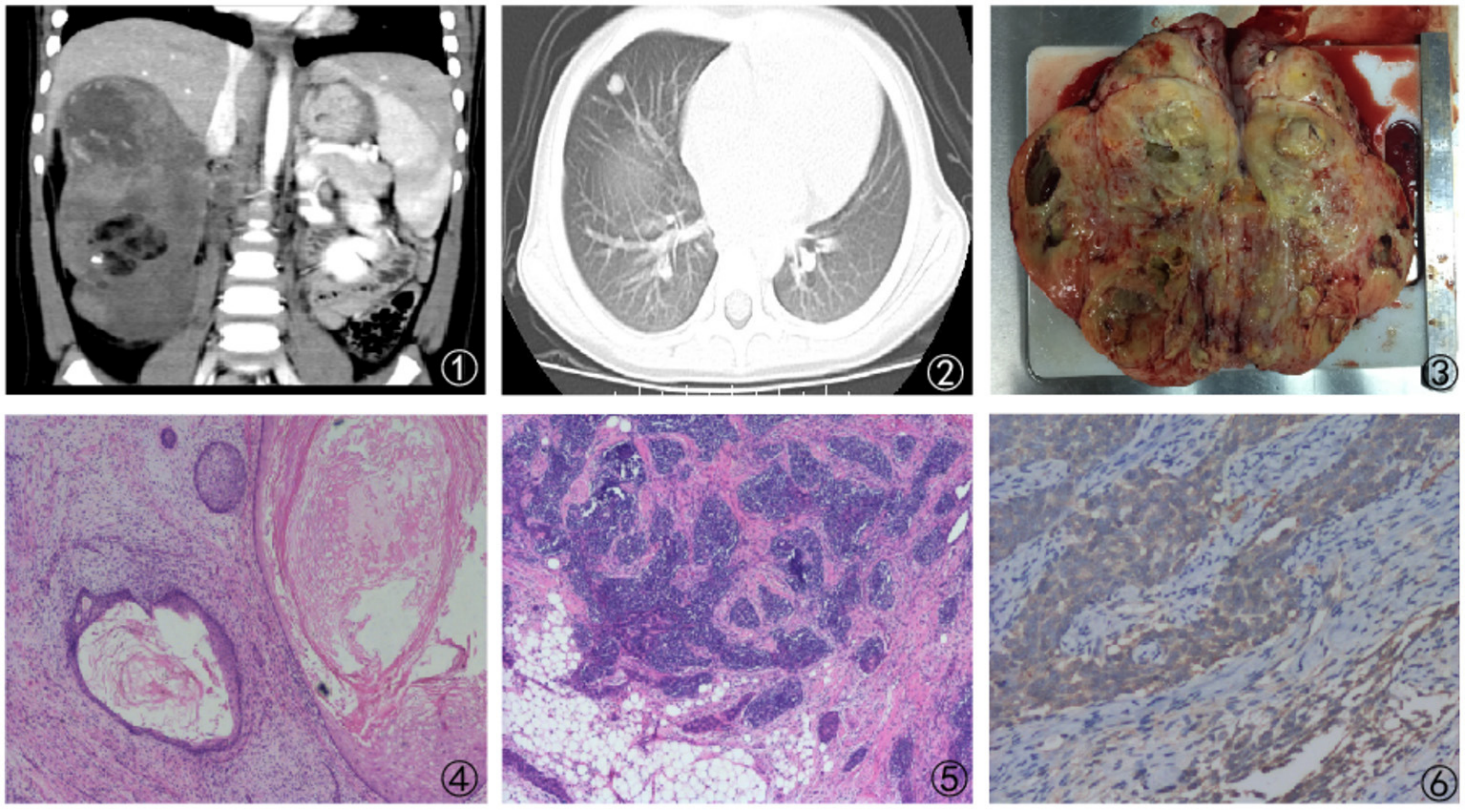
Findings from a 38-month-old girl presenting with abdominal pain for 1 week. (1) Abdominal CT scan revealing a large mass that had almost fully replaced the right kidney; (2) Lung metastases as revealed by the presents of a large lesion in the right lung; (3) Cross-sectional surface of the kidney, exhibiting a large mass that had largely replaced the normal tissue compartment; (4, 5) Imaging findings revealing prominent heterologous components that include squamous epithelial cells; (6) Positive WT-1 immunostaining in intratumoral tubules.

The next case was a 38-month-old female with a 1-week experience of abdominal pain, and abdominal ultrasonography revealed a solid right renal tumor (7 cm × 8 cm × 9 cm) exhibiting microcalcification. Abdominal CT scans demonstrated the presence of a large heterogeneous mass in the right renal posterior region (8 cm × 8 cm × 9 cm, Figure 3), with this tumor extending further on the cortex without crossing over the midline. The patient did not exhibit any evidence of bone marrow, pulmonary, or abdominal metastases and underwent right nephrectomy, yielding a 350 g mass. Histological analyses revealed this tumor to harbor regions with classical WT-like morphology admixed with regions of keratinized squamous cells in the form of discrete epidermoid cyst-like nodules composing >70% of the tumor volume. The patient underwent four cycles of postoperative chemotherapeutic treatment and was discharged from the hospital 10 years previously. The patient remains in good health without relapse (Figure 4).

#### Case 5

A 3-year-old girl came to our hospital with a left-sided abdominal mass that had been present since 2 months of age and a low-grade fever that had been present for 2 weeks. The blood pressure was normal at the time of presentation. In the left lumbar region, an 8 cm^3^ × 9 cm^3^ × 9 cm^3^ mass was palpable. This mass did not cross the midline and was not correlated with any other abnormalities. Urinary analyses revealed evidence of microscopic hematuria and sterile culture findings. Hemoglobin levels of the patient were 8.7 gm/dl. Renal and chest radiographic findings were normal, as were liver function tests. Abdominal ultrasonography revealed a large heteroechoic mass (10 cm × 10 cm × 9 cm) extending from the lower pole of the left kidney. Right kidney of the patient was normal, and no blood vessels were observed. Contrast-enhanced abdominal CT scans confirmed the presence of a heterogeneous mass (10 cm × 10 cm × 9 cm) extending from the middle and lesser poles of the left kidney. Good excretion of contrast with splaying of pelvicalyceal system was seen. There was no enlargement of local lymph nodes. Laparotomy was performed *via* a supraumbilical transverse transperitoneal incision, and a left radical nephrectomy was conducted. Examination of the contralateral kidney revealed it to be normal. The patient was doing well as of a 6-month follow-up.

## Discussion

One of the most prevalent forms of primary renal tumors in children is WT. These tumors exhibit characteristic features including efforts to recapitulate various stages of the nephrogenic process, and harbor blastemal, stromal, and epithelial cell types in a triphasic combination. In some cases, heterotopic mesodermal elements are also evident, representing a small proportion of the overall tumor. Atypical renal tumor times, i.e., TWT, clear cell kidney sarcomas, rhabdoid kidney tumors, multilocular cystic nephromas, and renal teratomas, are relatively rare (35). TWT tumors exhibit the characteristics of a diverse array of cell categories and tissues in addition to areas with classical nephroblastoma characteristics, and are diagnosed when >50% of the solid tumor exhibits the clear predominance of teratoid elements. In previously published reports, these TWTs have been reported to include a range of elements, such as neurological tissues, skeletal muscle, mucinous epithelial tissue, adipose tissue, squamous epithelial cysts, and cartilage (27, 28). Herein, differences in the volume of the tumor were not significant, in large part because of the very small size and n SD values in the TWT group. However, tumor volumes were substantially larger for patients with TWT relative to those in classical patients with WT, despite similar tumor weight values in both groups. This thus suggests that the density of TWT tumors may be lesser compared to classical WT tumors, potentially owing to the unique adipose tissue and squamous epithelial cysts present within TWTs.

Wilms tumor can arise as a consequence of abnormal genetic changes in the 11p13 (WT1) and 11p15 (WT2) regions of the short arm of chromosome 11. In nine described TWT cases identified to date, monosomy 11 or 11p deletion has been detected (35). The WT1 gene encoded in the 11p13 region is composed of 10 exons spread over a 50 kb region that gives rise to a 3 kb mRNA. The carboxyl-terminal region of WT1 (exons 7–10) consists of four zinc finger motifs that give rise to a DNA-binding domain. This domain enables WT1 to bind to corresponding promoter sequences in downstream target genes, and mutations within this domain can alter this DNA-binding activity to adversely impact WT1 function (10). Ghamdi et al. reported on the presence of germline *WT1* gene mutations in children that were predisposed to WT in a mutational analysis, although they specifically focused on tumor tissue samples. In line with reported *WT1* mutation rates in sporadic WT cases, only one of the three cases exhibited a *WT1* mutation. There have been few studies of *WT1* gene mutations in TWT to date, and available evidence suggests that these findings are comparable to those in classical WT with mutations only being evident in a small subset of patients (36). Interestingly, three of our patients exhibited WT1 positivity in immunohistochemical staining assays, suggesting WT1 may offer potential value in this context, although further genetic analyses will be needed to further clarify this issue.

In general, TWT is considered to be a relatively non-aggressive tumor type to be relatively chemoresistant and radioresistant owing to its differentiated teratomatous composition. However, it is possible that these assumptions may be inaccurate. Treatment strategies for TWT have yet to be determined owing to the scarcity of this tumor type and its varied composition. To date, four patients with TWT who have died have been reported to date, with two dying of metastatic disease whereas the third died of renal failure and sepsis (7, 37). Regarding the considered cases, two exhibited metastases and one experienced postoperative pelvic recurrence potentially related to needle biopsy. These tumors may thus exhibit aggressive features, such as anaplasia and regional or distant metastasis, with anaplastic features contributing to the risk of such metastasis and associated mortality. To further establish the relative malignancy of TWT, further research is warranted in an effort to establish more appropriate and reliable treatment guidelines.

This study has several limitations. A relatively small sample size of children with TWT, patients originating from one hospital, and a retrospective study design constituted the limitations of this study. Despite these limitations, this was the first study to evaluate TWT in the context of WT, which may provide new insights in a previously unknown continent.

## Conclusion

In summary, our patients with WT are the first reported cases of TWT in Chinese children to date. These preliminary results suggest that the tumors in patients with TWT may be less dense than those in patients with classical WT. Based on these findings, we recommend the treatment of this rare tumor type with specific protocols. There is additionally a clear need to conduct more basic research aimed at clarifying the biological nature of this tumor type and to identify appropriate theranostic markers that can be leveraged to optimize individualized treatment strategies for affected patients. Genetic studies also have the potential to clarify whether there are specific biomarkers associated with this cancer type. Owing to the highly differentiated and varied nature of these tumors, however, they are likely to respond poorly to chemotherapy, although we believe that surgical treatment alone is unsatisfactory as a means of treating this scarce tumor type.

## Data Availability Statement

The raw data supporting the conclusions of this article will be made available by the authors, without undue reservation.

## Ethics Statement

The studies involving human participants were reviewed and approved by the Ethics Committee of Shanghai Children's Hospital, Shanghai Jiao Tong University. Written informed consent to participate in this study was provided by the participants' legal guardian/next of kin. Written informed consent was obtained from the individual(s), and minor(s)' legal guardian/next of kin, for the publication of any potentially identifiable images or data included in this article.

## Author Contributions

WW and YW contributed to the study design. JL and WX contributed to data collection. WW and JL contributed to the analysis of data. WW and ZL contributed to manuscript writing. All authors approved the submitted version.

## Funding

This study was funded by the Shanghai Health Committee (20214Y0476).

## Conflict of Interest

The authors declare that the research was conducted in the absence of any commercial or financial relationships that could be construed as a potential conflict of interest.

## Publisher's Note

All claims expressed in this article are solely those of the authors and do not necessarily represent those of their affiliated organizations, or those of the publisher, the editors and the reviewers. Any product that may be evaluated in this article, or claim that may be made by its manufacturer, is not guaranteed or endorsed by the publisher.

## Abbreviations

WT: Wilms tumor
TWT: teratoid Wilms tumor.
